# Epstein–Barr Virus (EBV) and Multiple Sclerosis Disease: A Biomedical Diagnosis

**DOI:** 10.1155/2022/3762892

**Published:** 2022-08-18

**Authors:** Asma Alanazi

**Affiliations:** ^1^Department of Basic Medical Sciences, College of Medicine, King Saud Bin Abdulaziz University for Health Sciences, Riyadh, Saudi Arabia; ^2^King Abdullah International Medical Research Centre (KAIMRC), Riyadh, Saudi Arabia

## Abstract

Multiple sclerosis (MS) is a degenerative disease that affects 2.8 million people worldwide. It is a central nervous system disease (CNS), in which the myelin sheath covering the brain and spinal cord neurons is attacked. If the myelin sheath is damaged, a person can suffer permanent damage to the nerves. There are a number of factors that can increase a person's risk of developing MS, such as obesity, smoking, vitamin D deficiency, certain tissue types (HLADRB1*∗*15 : 01) and infection with the Epstein–Barr virus (EBV). The latter virus can cause infectious mononucleosis, which can, in turn, result in lifelong infection in the host. To establish the relationship between MS and EBV, the author conducted a study on 1176 MS patients admitted to Saudi Arabia King Abdulaziz City centers. The researcher determined that MS occurred twice as much in females as it did in males, and also that EBV was much more widespread in MS female patients than MS male patients (27 : 1). Age was not a factor in the occurrence of EBV. There were limitations on data completeness and availability. Other trials using larger cohorts of patients are needed.

## 1. Introduction

Multiple sclerosis (MS) is a chronic inflammatory disease impacting the central nervous system (CNS) and one of the leading causes of neurological disability in adults. It is caused by the immune cells that attack CNS-self cells as if they were foreign bodies, which causes demyelination and neurodegeneration in the CNS. The damage leads to a gradual deterioration of sensory, motor, and cognitive functions [1, 2]. Although there are various forms of MS, the disease typically takes the form of relapsing-remitting (RR) cycles of disease activity. In rare cases, primary-progressive (PP) disease occurs. It is common for RRMS to progress over time and become a secondary-progressive (SP) disease [3]. Not everyone is susceptible to MS; the host must be genetically susceptible in the HLA-II locus after interaction with environmental factors [4]. MS arises from interactions between an individual's genetic susceptibility and environmental factors. One of the most MS-associated susceptibility alleles is HLA DRB1*∗*1501 [5]. Moreover, there are several environmental risk factors that can increase a person's risk of developing MS, such as exposure to sunlight/high vitamin D levels, smoking, and infectious agents [1]. One of the most common infectious agents that causes the condition is the Epstein–Barr virus (EBV), a human DNA herpes virus that has a strong connection with MS [6, 7].

Research shows that EBV is one of the world's most well-developed human viruses. It is usually spread through saliva. In most infected people, EBV creates an asymptomatic, lifelong infection which is carried as an infection of B lymphocytes for the duration of one's life. The virus, however, is controlled by the host's immune system. It can–very rarely though–cause infectious mononucleosis and human tumor malignancies, such as lymphomas and nasopharyngeal carcinoma [8]. The rare occasions detailed earlier are due to a virus-host imbalance, one that brings about the pathogenic potential of EBV [9, 10].

Two studies conducted in Gulf Cooperation Council countries, as well as one study in Kuwait and a further study, have examined the relationship between EBV and MS. used data obtained from US military recruits over a period of 20 years between 1993 and 2013 [11]. The studies had conflicting reports. One study in Kuwait was epidemiological and did not establish an association between EBV and MS, while the other one in Kuwait was a serological study that showed a strong link between EBV and MS [12]. What's more, [11] found the risk of developing MS is largely increased by contracting EBV infection. Moreover, it was found that this virus often precedes the onset of MS, which further supports the belief that it may play a role in MS pathogenesis. The study involved over 10 million young adults working for the US army. Risks of MS increased 32-fold post-EBV infection, but not other viruses of similar infection method, such as the cytomegalovirus. The findings showed that, after EBV seroconversion occurred, there was an increase in the serum levels of the neurofilament light chain, which is a critical indicator of neuroaxonal degeneration. Other than the presented studies, a number of large-scale epidemiological and serological studies in Western countries showed that EBV is strongly associated with MS [13], evidence being virtually 100% EBV seroprevalence in adult MS, and higher anti-EBV antibody titers, specifically for EBNA1 IgG in MS patients [7, 14], and an increased risk of developing MS after infectious mononucleosis [15–17]. Infectious mononucleosis, a feature of EBV pathology, more than doubles the risk of MS.

In EBV-infected individuals, the immune reactivity toward the virus is higher in people with MS, indicating inadequate control of the virus [15]. Meanwhile, evidence from neuropathological and immunological studies indicates that continual EBV infection in the central nervous system can generate an immunopathological CD8 T-cell response in order to eliminate the virus. However, this ultimately results in bystander CNS injury [18]. We searched for EBV-specific CD8 T cells in MS brain tissue. The latter researchers examined the postmortem brain samples of 12 donors who had progressive MS and had been diagnosed with the HLA class 1 genotype [19]. Their work concluded that the expression of CD8 cytotoxic T cells was detected toward the expression of proteins in the latent and lytic phases of the EBV life cycle. In addition, they determined that these immune cells were detected in the white matter lesions and/or meninges of 11–12 MS donors. The T cells were also found to be adhered to EBV-infected cells. The median value of CD8 T cells recognizing individual EBV epitotes fell in the range of 0.5 to 2.5% of CNS-infiltrating CD8 T cells. CNS-infiltrating EBV-specific CD8 T cells showed a cytotoxic phenotype as they were CD107a positive. Additionally, the relationship between EBV-related immune responses and CNS-related inflammation that causes MS pathology is supported by the presence of local EBV dysregulation and selective enrichment of EBV-specific CD8 T cells in the brains of MS sufferers. the prevalence of MS has increased in Saudi Arabia, and there is no study in Saudi Arabia evaluating the correlation between EBV and MS. Therefore, the aim of the study is to assess the association between EBV and MS patients in King Abdulaziz medical centers from 2015 to 2022.

## 2. Methods

**Figure 1:**
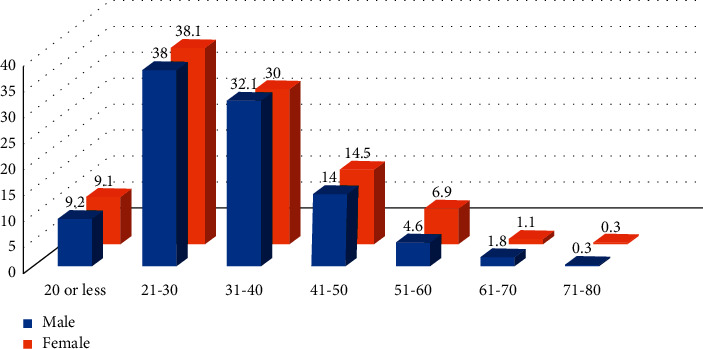
Distribution of MS by age group in a sample of 1176 patients.

**Figure 2:**
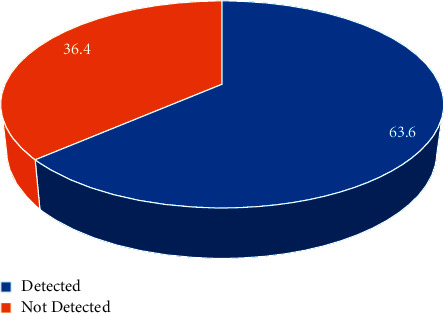
Percent of EBV detected in a sample of 44 MS patients.

A descriptive cross-sectional study was carried out among 1176 MS patients whose electronic medical records were kept in King Abdulaziz City Centers for the admittance years of 2015 to 2022. The data were obtained by the BEST Care system. Data were coded for entry and analysis using SPSS statistical software (version 22.0). The research earned the approval of the Institutional Review Board (IRB) from King Abdullah International Medical Research (KAIMRC). IRB^#^ NRC22R/102/02. The demographic characteristics included frequency, percentage, mean, and standard deviation. For nonparametric risk factors, such as gender and symptoms, chi-square test was utilized. A one sample *t*-test was used to test the significance of the mean of interval and ratio variables, such as age as shown in Figures 1 and 2.

## 3. Results

Multiple sclerosis was as twice in the female population as in the male cohort in the sample. There was no statistically significant difference between males and females regarding the distribution of the disease by age group (*P*=0.432). The mean age of female was 32.95 ± 10.762 and the mean age of males was 33.12 ± 10.791 as shown in Table 1.

In a subsample of 44 patients, the percentage of detected Epstein–Barr virus was 63 percent.

There was a statistically significant difference between males and females regarding the number of detected cases of EBV. The virus was detected in all women (100%) while it was only detected in 5.9% of males as shown in Table 2.

Epstein–Barr virus IgM was detected in more than one third of the subsample (35.7%). EBV-IgG was detected in 28.6% while Epstein–Barr virus PCR (Quantitative) was only detected in 3.6% of the sample. The average for EDSS score was 4.02 for the subgroups. The EDSS scale ranges from 0 to 10 in 0.5 unit increments that represent higher levels of disability. The EDSS scale varies between 0 and 10 in 0.5 unit increments, each of which denotes a higher level of disability as shown in Table 3.

## 4. Discussion

The author conducted this study by using electronic medical records in King Abdulaziz City for the years 2015–2022 on the number of MS patients and the cases of EBV detected. There were 1176 MS patients with females comprising 66.7% of the sample (*P* = 0.432), twice as much as the male cohort. Genetic factors are most likely to blame for the female preponderance of MS. The mean age of the female cohort (32.95 ± 10.762) overlapped with that of the male cohort (33.12 ± 10.791). In a sample of 44 MS patients who were on disease-modifying therapies (DMTs), EBV was detected in 28 of them. Only 1 male had EBV while the rest of the sample consisted only of females. In these 28 cases, IgM was detected in 35.7% of the samples while EBV PCR was only detected in 3.6% of the samples. EBV is known for its latent course of infection. About 95% of the population gets this virus during childhood [20]. Seroconversion from negative to positive for EBV antibodies increases with age. The incidence peaks early in childhood and again around puberty, especially for females. This is coincident with the female predominance in MS [21,22]. However, it is possible for the virus to reactivate in some instances. This is not always accompanied by symptoms. However, symptoms are more likely to develop in individuals with compromised immune systems (i.e., those with MS).

The primary limitation of the current study is that the study population only included patients admitted to NGHA, Riyadh, Saudi Arabia.

## Figures and Tables

**Table 1:** Comparison between gender age of the sample of MS patients (*n* = 1176).

Variables	Number	Percent	Number	Percent
Gender				
	Male		Female	
	392	33.3	784	66.7

Age				
20 or less 21–30 31–40 41–50 51–60 61–70 71–80	36 149 126 55 18 7 1	9.2 38.0 32.1 14.0 4.6 1.8 0.3	71 299 235 11454 9 2	9.1 38.1 30.0 14.5 6.9 1.1 0.3

Mean sd	32.95 ± 10.762		33.12 ± 10.791	

Independent *T* test		*F* = 0.681	d*f* = 1174	*P*=0.432

**Table 2:** Comparison between Male and Female in the MS-EBV cohort (*n* = 28).

	Male		Female		Chi square value	*P*
	Number	Percent	Number	Percent		
Detected	1	5.9	27	100	39.933	0.001
Not detected	16	94.1	0	0		

**Table 3:** Type of test used for detected cases (*n* = 28).

Test type	Frequency	Percent
EBNA antigen IgG	6	21.4
EBV-IgG	8	28.6
Epstein–Barr virus IgM	10	35.7
Epstein–Barr virus nuclear antigen IgG antibody	3	10.7
Epstein–Barr virus polymerase chain reaction (PCR) (quantitative)	1	3.6
Total	28	100.0

## Data Availability

The data used to support the findings of this study are included within the article.
